# Relationship between nutrition knowledge level, body perception and emotional eating among pregnant and lactating women: a cross-sectional study of 200 Turkish women

**DOI:** 10.1186/s12884-025-08622-9

**Published:** 2026-01-24

**Authors:** Hatice Kübra Barcin Güzeldere, Şevval Gençtürk, Zeynep Türker, Ergül Demirçivi, Bashir A. Lwaleed

**Affiliations:** 1 Department of Nutrition and Dietetic, Istanbul Medeniyet University, Istanbul, Türkiye Turkey; 2 School of Health Sciences, University of Southampton, South Academic and Pathology Block (MP 11), Southampton General Hospital, Tremona Road, Southampton, SO16 6YD UK; 3 Department of Gynecology and Obstetrics, Göztepe Prof. Dr. Süleyman Yalçın Hospital, Istanbul, Turkey

**Keywords:** Body image, Emotional eating, Lactation, Nutritional knowledge, Pregnancy

## Abstract

**Background:**

Nutrition knowledge is crucial for a healthy pregnancy and lactation period. Misleading information about healthy nutrition can lead to health problems. Therefore, it is important to obtain information about healthy nutrition from the right sources and increase awareness of healthy nutrition, especially during these periods. This study aims to explore the relationships among nutritional knowledge level, body perception, and emotional eating among pregnant and lactating women.

**Methods:**

A total of 200 pregnant and lactating women (100 participants each) were included. A questionnaire was created by researchers, and it consists of 5 sections covering demographic data, the Nutrition Knowledge Level Determination Scale for Adults (NKLSA), false facts about nutrition during pregnancy and lactation (FFNPL), the Photographic Figure Rating Scale (PFRS), and the Emotional Eating Questionnaire (EEQ). All the data were collected face to face from a single-center gynecology department.

**Results:**

Pregnant and lactating women had similar nutritional knowledge levels (*p* > 0.05). Basic nutritional knowledge was associated with emotional eating and FFNPL. The majority of pregnant and lactating women had moderate knowledge about basic nutrition and low emotional eating, which was particularly significant for lactating women (pregnancy *r* = 0.288 *p* > 0.05, lactation *r* = 0.130 *p* < 0.05).

**Conclusion:**

The study findings suggest that pregnant and lactating women experience emotional eating and distorted body perception, which are linked to their nutritional knowledge levels. To improve nutritional knowledge among pregnant and lactating women, public health strategies, such as healthy nutrition educational programs should be planned.

**Trial registration:**

There is no need for trial registration.

**Supplementary Information:**

The online version contains supplementary material available at 10.1186/s12884-025-08622-9.

## Background

Healthy, adequate, and balanced nutrition is important, especially during pregnancy and lactation. The nutritional needs of the mother, fetus, and neonate must be met during this period. The promotion and maintenance of health are closely associated with nutrition. Energy and nutrient requirements need to increase during pregnancy to support fetal life and milk production during breastfeeding [1]. Adequate intake of nutrients is linked to lower rates of pregnancy-related and neonatal problems [2]. After birth, breastfeeding is also influenced by maternal nutrition. While the quantity of milk is important, its quality—especially fat content—varies with the nutritional status of the mother [3]. These nutrients include iron, folate, calcium, vitamin D, and omega-3 fatty acids, which are especially important for the physical and physiological health of newborns [1]. Studies have shown that unhealthy food preferences can adversely affect fetal growth, a critical indicator of a baby’s health [4–6]. A sugar-rich diet is associated with low birth weight among newborns, whereas a diet rich in seafood, vegetables, and fruits is linked to higher birth weight among newborns [6].

Despite established guidelines for adequate nutrient intake, pregnant and lactating women often struggle to meet these recommendations [3, 7, 8]. Importantly, an inadequate diet can lead to nutritional deficiencies. Sufficient nutrient intake is achieved through a healthy, adequate, and balanced diet. Food preferences play a key role in healthy nutrition and are influenced by various factors during pregnancy and lactation [9]. Research indicates that knowledge about nutrition significantly impacts diet quality and food preferences. Understanding healthy foods is a crucial first step in adapting to a healthy diet [10]. Moreover, nutritional knowledge can lead to behavioral changes [11]. Various studies have examined nutritional knowledge during pregnancy and lactation, focusing on aspects such as food sources of folate, the use of folic acid supplements, weight gain recommendations, and daily serving sizes [12]. Conversely, misconceptions about nutrition during this period can adversely affect food intake [13]. Research has shown that pregnant women tend to rely on friends and relatives as information sources about nutrition rather than seeking advice from health professionals who are experts in the field [14, 15]. Although women during pregnancy and lactation possess a certain level of awareness about nutrition, it appears that the information they obtain is often from unreliable sources [16]. Research shows that nutrition education programs that aim to improve nutritional knowledge and breastfeeding practices encourage women to maintain breastfeeding and improve healthy nutrition [17–19]. In particular, lactating women face problems in terms of cultural beliefs [20]. The nutritional education background and high awareness of nutrition are linked with proper nutritional practices [21].

In addition to nutritional knowledge, changes in hormonal balance during pregnancy and lactation, increased stress, and unhealthy eating habits can influence emotional eating [22]. Emotional eating is defined as the tendency to overeat in response to negative emotions, such as anxiety or irritability [23]. These emotion-based changes in eating behavior can range from overeating to severe calorie restriction [24]. Excessive weight gain caused by binge eating can lead to numerous adverse pregnancy outcomes, such as increased gestational weight gain, an increased cesarean section rate, early onset of obesity, and macrosomia [25]. Emotional eating can be triggered by food cravings, which are especially common during pregnancy and may result in obesity due to additional weight gain [22, 26]. Fariandini et al. [27] reported that, owing to emotional fluctuations, pregnant women can struggle to control their appetite, which changes with mood and concludes with emotional eating. In contrast, Köse et al. [28] reported that lactating women can be influenced by the social environment, which can help them make good choices for healthy nutritional habits. However, this stage can change over time and lead to emotional eating due to the accumulation of stressors [29]. Pickett et al. [30] reported that emotional eating often leads to the consumption of high-calorie, palatable foods in response to negative emotions, reinforcing the cycle of emotional distress and unhealthy eating habits. In addition, changes in ovarian hormone levels trigger emotional eating toward excessive eating behaviors with the help of stressors such as pregnancy and motherhood [31, 32].

On the other hand, due to changes in hormones, body dissatisfaction commonly occurs in pregnant and lactating women. Body image reflects people’s positive or negative thoughts and perceptions about their bodies and appearance, encompassing their attitudes toward themselves [33]. The body changes associated with pregnancy and lactation can negatively affect the body image of pregnant and lactating women [34]. A common concern among pregnant women is the fear of not returning to their pre-pregnancy physical form, which can lead to body image problems. In addition, unrealistic beauty standards exacerbate pregnant and lactating women in terms of dissatisfaction [35]. Studies support the notion that various psychological concerns, including body image dissatisfaction, particularly increase during the postpartum period [36–38]. Some studies indicate that pregnant women experience greater dissatisfaction with their appearance during pregnancy than before [36–38]. This dissatisfaction tends to peak in the early stages of pregnancy [39]. Pregnancy and lactation affect the skin, hair, abdominal area, hips, and breasts due to increased fat storage [40]. Additionally, research suggests that unhealthy nutrition and negative eating behaviors during pregnancy may be associated with concerns about body image [41]. The relationship between nutritional knowledge, body dissatisfaction, misconceptions about nutrition and emotional eating among pregnant and lactating women have not been well-known. Due to this gap, this study aims to evaluate the relationships among nutritional knowledge, emotional eating, misconceptions about nutrition and body image during pregnancy and lactation among Turkish women.

## Methods

### Participants

A total of 200 Turkish women participated in the study, with 100 participants each in the pregnancy and lactation groups. The exclusion criteria included individuals with eating disorders, psychological disorders (such as depression, schizophrenia, bipolar disorder), and diabetes. The exclusion criteria of participants were determined according to their self-reported medical and behavioral information. The inclusion criteria determined as:

- Healthy pregnant women.
- Currently healthy breastfeeding women (0–24 months).
- Women without gestational diabetes for pregnant women.
- Women without type 2 diabetes for lactating women.
- Healthy pregnant or lactating women who had an appointment for routine check-ups with a gynecologist.
- Women who has no eating or psychologic disorder. Only individuals who had been examined in the hospital Obstetrics and Gynaecology Department for routine check-ups and whose clinical information was available in the hospital database were included in the study. The survey form, created by the researchers, was filled out face-to-face through a question-and-answer format with eligible participants who voluntarily agreed to participate in the study. The study sample size was calculated using G*Power 3.2, with an effect size (d) of 0.2, a 5% error rate (α), and a test power of 80% (1-β) based on the results of two studies on the nutritional knowledge of healthy pregnant and lactating women [42, 43]. A total sample size of 194 (97 for each) was determined to be adequate for the study. Ethical permission was obtained from Istanbul Medeniyet University Göztepe Training and Research Hospital Clinical Research Ethics Board (Reference No: 2022/0711). Data were collected from September 2022 to March 2023 at a single center (Göztepe Prof. Dr. Süleyman Yalçın City Hospital, Obstetrics and Gynecology Department). The hospital is a primary care hospital, and annually provides services to approximately 1.8 million outpatients and 600,000 emergency department visitors, in addition to delivering inpatient care to nearly 50,000 individuals. Moreover, the institution performs approximately 60,000 surgical procedures each year across categories A, B, C, D, and E. All data collected face-to-face at hospital. All research was conducted under the terms of the Declarations of HELSINKI.

### The survey

The data were collected via a face‒to-face survey consisting of five parts created by the researchers. The survey contained a total of 93 questions. The first part of the survey included demographic data (age in years, weight (kg), height (cm), weight gained during pregnancy (kg), gestational age (years), gestational week/lactation status, lactation period, disease status, self-declarated economic status, and cravings during pregnancy). The second part assessed the Nutrition Knowledge Level Determination Scale for Adults (NKLSA). The third part included questions addressing common misconceptions about nutrition (FFNPLQ). The data for these questions were sourced from websites accessed via keywords such as “nutrition during pregnancy,” “milk-enhancing foods/drinks,” “what not to eat during pregnancy,” and “increasing the baby’s weight during pregnancy.” The fourth section utilized the Photographic Figure Rating Scale (PFRS) to evaluate body image, whereas the fifth and final sections included the Emotional Eating Questionnaire (EEQ) to assess emotional eating. The survey presented in supplementary material. Permissions to use all measurement scales included in this study were formally obtained from their developers or authorized distributors.

### The nutrition knowledge level determination scale for adults (NKLS)

The scale was developed by Hilal Batmaz and Fatma Esra Güneş [44] and consists of two parts: “Basic Nutrition Information” and “Food-Preference Information.” The basic nutrition information section comprises a total of 20 questions, whereas the food-preference section contains 12 questions. The responses are classified via a 5-point Likert scale (“strongly agree,” “agree,” “neither agree nor disagree,” “disagree,” and “strongly disagree”). For the evaluation, responses are allocated points as follows: strongly agree = 0 points; agree = 1 point; neither agree nor disagree = 2 points; disagree = 3 points; and strongly disagree = 4 points for items 1, 3, 6, 8, 13, 16, 19, and 20 in the “Basic Nutrition Knowledge” section. Other items are scored inversely. In the food preference section, all the items except for items 8 and 10 are reverse-scored. The participants’ knowledge levels in the basic nutrition section were classified as follows: less than 45 points = poor, 45–55 points = moderate, 56–65 points = good, and above 65 points = very good. In the food preference section, knowledge levels are classified as follows: less than 30 points = poor, 30–36 points = moderate, 37–42 points = good, and above 42 points = very good. The reliability of the NKLS was found as follows: the internal reliability coefficient of the 20 propositions under the title of “Basic nutrition” was Cronbach’s Alpha = 0.72, and the internal reliability coefficient of the 12 propositions under the title of “Food preference” was found as Cronbach’s Alpha = 0.74.

### Creating false facts during pregnancy and lactation questionnaire (FFPLQ)

In the survey, questions about common false facts related to nutrition during pregnancy and lactation was developed by the researchers and were included in the third section of the survey form (supplementary material). The questionnaire were derived from a systematic review of the most frequently asked nutrition-related questions by pregnant and lactating women on reputable health websites and public platforms [45–63]. This section consists of 25 questions, with answers framed as “yes” or “no.” The correct answers to questions 2, 5, 10, 11, 15, 16, 17, 18, and 19 are “YES,” whereas the correct answers to the remaining questions are “NO.” Each question has a score of 4 points. Participants scoring less than 32 points from the sum of correct responses are classified as having poor knowledge, scores between 36 and 72 are considered moderate, and scores of 76 and above indicate a high knowledge level. The questionnaire was approved by a lecturer at the Istanbul Medeniyet University Department of Nutrition and Dietetics.

### Photographic figure rating scale (PFRS)

The scale was developed by Swami, Salem, Furnham, and Tove [64], and its Turkish validity and reliability studies were conducted by Sertel-Berk and Yücel [65]. The scale ranks 10 female figures from underweight to obese on the basis of body mass index (BMI) categories: Fig. 1: BMI 13.616 and below; Fig. 2: BMI 13.617–15.685; Fig. 3: BMI 15.686–17.55; Fig. 4: BMI 17.56–19.39; Fig. 5: BMI 19.40–21.71; Fig. 6: BMI 21.72–25.015; Fig. 7: BMI 25.016–28.10; Fig. 8: BMI 28.11–32.59; Fig. 9: BMI 32.60–38.57; Fig. 10: BMI 38.58 and above. There are a total of 5 questions on the scale. Questions 1, 2, and 3 pertain to physical attractiveness, whereas Questions 4 and 5 focus on body dissatisfaction. Within the scope of construct validity of the scale, its correlation with the Body Image Satisfaction Scale was found to be −0.31 and its test-retest reliability was found to be 0.83.

#### Body dissatisfaction (PFRS-BD) score

The respondent’s answer to Question 4 is divided by their answer to Question 5, and the result is multiplied by 100. If the result is below 100, the participant perceives themselves as thin; if it is above 100, they perceive themselves as overweight, indicating body dissatisfaction. A score of exactly 100 suggests that the participant does not perceive any dissatisfaction with their body.

#### Body image disturbance (PFRS-BID) score

Each participant’s answer to Question 4 was divided by the number corresponding to their BMI, and the result was multiplied by 100. If this result is less than 100, it indicates that the participant perceives themselves as lighter than they actually are, suggesting a distorted body image. If the result is above 100, it indicates a concern of body image disorder. A score of 100 indicates that the participant does not have issues with body image.

### Emotional eating questionnaire (EEQ)

The scale was created by Garaulet et al. [66], and the Turkish adaptation was developed by Arslantaş et al. [67]. This scale consists of 10 items organized into three categories: the inability to resist the desire to eat, types of foods, and feelings of guilt. The questions are of a Likert type with 4 response options (“0” Never, “1” Sometimes, “2” Usually, and “3” Always). The lowest possible score is “0,” whereas the highest is “30.” The cutoff score is set at 21. According to the creators, a score of “0–5” indicates “not an emotional eater,” a score of “6–10” indicates a “low-level emotional eater,” a score of “11–20” indicates an “emotional eater,” and a score of “21–30” suggests a “hyperemotional eater.” Within the scope of the Reliability Analysis of the Emotional Eating Scale, the Cronbach alpha internal consistency coefficient was calculated as 0.84 (95% CI: 0.82–0.86).

### Statistical analysis

The data were analyzed via SPSS 26. The normality of the data was assessed via the Kolmogorov‒Smirnov test, Shapiro‒Wilk test, skewness‒kurtosis values, and histograms. Descriptive variables are presented as the means ± standard deviations (SDs) and percentages (%). The scale results of the participants were compared via the “t test for independent samples” for normally distributed two groups and the “Mann‒Whitney U test” for nonnormally distributed data. A bivariate correlation method was employed to determine the relationships between body image, nutritional knowledge level, and emotional eating. Predictors for questionnaire scores were identified via linear regression analysis. Differences were considered significant at *p* < 0.05.

## Results

The demographic variables of the study participants are presented in Table 1. The mean age of the pregnant women was 29.12 ± 5.64 years, while the mean age of the lactating women was 29.92 ± 5.42 years (*p* > 0.05). Furthermore, all participants presented similar anthropometric data, as well as comparable educational, economic, and health statuses (*p* > 0.05).

**Table 1:** Demographic variables of the participants

	Pregnant (*n* = 100)	Lactate (*n* = 100)	*P*-value
	Mean ± SD	Mean ± SD	
Age (years)	29.12 ± 5.64	29.92 ± 5.42	0.308
Gestational age/Lactating period	28.63 ± 9.63	3.03 ± 4.28	-
Weight (kg)	75.05 ± 19.38	74.07 ± 13.38	0.676
Height (cm)	162.28 ± 5.13	162.20 ± 6.57	0.924
	**% (n)**	**% (n)**	
Education			0.288
İlliterate	0 (0)	3 (3)	
Primary school	10 (10)	5 (5)	
Secondary school	22 (22)	24 (24)	
High school	31 (31)	28 (28)	
Graduate	35 (35)	35 (35)	
Master	2 (2)	5 (5)	
Economic status			0.619
Low	8 (8)	10 (10)	
Moderate	71 (71)	74 (74)	
High	21 (21)	16 (16)	
Do you have any disease?			
Yes	77 (77)	82 (82)	0.396
No	23 (23)	18 (18)	
Did you experience cravings during your pregnancy?			
Yes	64 (64)	63 (63)	0.883
No	36 (36)	37 (37)	
Which meals/foods/drinks do your cravings for during your pregnancy? *			
Sugar added foods (chocolate, cookie, cakes etc.)	44.2 (23)	48.3 (28)	-
Sugar added beverages (fruit juice, hot chocolate, iced tea, lemonade etc.)	25 (13)	13.8 (8)	
Carbonated drinks (coke, lemonade, spring waters)	21.2 (11)	20.7 (12)	
Fresh fruit juices	26.9 (14)	12.1 (7)	
Fast foods (hamburger, pizza, doner kebab etc.)	30.8 (16)	22.4 (13)	
Sour fruits (peels, oranges, kiwi etc.)	59.6 (31)	48.3 (28)	
Sweet fruits (watermelon, melon, peach, strawberry etc.)	48.1 (52)	46.6 (27)	
Do you think that you are committed a healthy diet?			0.130
Yes	63 (63)	73 (73)	
No	37 (37)	27 (27)	
How many meals do you eat in a day?			
1	2 (2)	0 (0)	**0.047**^**⊗**^
2	10 (10)	21 (21)	
3	41 (41)	48 (48)	
4	23 (23)	14 (14)	
5	17 (17)	11 (11)	
6	7 (7)	4 (4)	
7<	0 (0)	2 (2)	
Which meals do you eat in a day? *			-
Breakfast	94 (94)	95 (95)	
Mid-morning snack	26 (26)	17 (17)	
Lunch	72 (72)	70 (70)	
Afternoon snack	45 (45)	37 (37)	
Dinner	98 (98)	95 (95)	
Night snack	29 (29)	21 (21)	
Which meals do you skip? *			
Breakfast	5.4 (5)	5.3 (5)	-
Mid-morning snack	80.6 (75)	88.3 (83)	
Lunch	29.0 (27)	31.9 (30)	
Afternoon snack	58.1 (54)	67.0 (63)	
Dinner	0 (0)	5.3 (5)	
Night snack	78.5 (93)	84.0 (94)	

*Multiple responses

^⊗^ chi-square test, significant at the *p* < 0.05 level

Table 2 presents the participants’ questionnaire scores. The analysis revealed that pregnant and lactating women had similar levels of nutritional knowledge (*p* > 0.05). Furthermore, both groups exhibited at least low levels of emotional eating behavior, with no significant difference between pregnant and lactating women (*p* > 0.05).

**Table 2:** Questionnaire scores of the participants

			Pregnant (*n* = 100)	Lactate (*n* = 100)	*P*-value
			Mean ± SD	Mean ± SD	
NKLSA- Basic Nutrition			49.07 ± 6.88	49.02 ± 6.21	0.957
NKLSA- Basic Nutrition			**% (n)**	**% (n)**	
	Poor		19 (19)	28 (28)	0.282
	Moderate		72 (72)	59 (59)	
	Good		8 (8)	11 (11)	
	Very good		1 (1)	2 (2)	
			**Mean ± SD**	**Mean ± SD**	
NKLSA- Food Preference			35.73 ± 6.19	36.12 ± 5.99	0.651
NKLSA- Food Preference			**% (n)**	**% (n)**	
	Poor		15 (15)	10 (10)	0.714
	Moderate		49 (49)	50 (50)	
	Good		24 (24)	25 (25)	
	Very good		12 (12)	15 (15)	
			**Mean ± SD**	**Mean ± SD**	
FFPLQ			69.56 ± 9.22	68.84 ± 9.77	0.593
FFPLQ			**% (n)**	**% (n)**	
	Poor		0 (0)	0 (0)	0.359
	Moderate		66 (66)	72 (72)	
	Good		34 (34)	28 (28)	
			**Mean ± SD**	**Mean ± SD**	
EEQ			7.88 ± 5.54	8.21 ± 4.99	0.659
EEQ			**% (n)**	**% (n)**	
	Nonemotional eater		39 (39)	35 (35)	0.712
	Low emotional eater		29 (29)	36 (36)	
	Emotional eater		30 (30)	28 (28)	
	Very emotional eater		2 (2)	1 (1)	
			**Mean ± SD**	**Mean ± SD**	
PFRS body dissatisfaction			222.01 ± 156.47	218.89 ± 148.08	0.370
PFRS body dissatisfaction			**% (n)**	**% (n)**	
	No body dissatisfaction		9 (9)	14 (14)	0.406
	Slimmer than what they were		8 (8)	5 (5)	
	Fatter than what they were		83 (83)	81 (81)	
			**Mean ± SD**	**Mean ± SD**	
PFRS body image disturbance			85.26 ± 29.64	82.12 ± 47.71	**0.047**^**σ**^
PFRS body image disturbance			**% (n)**	**% (n)**	
	No body image disorder		27 (27)	14 (14)	0.059
	Slimmer than what they were		59 (59)	73 (73)	
	Fatter than what they were		14 (14)	13 (13)	

_σ: Mann‒Whitney U test, *p*<0.05_

Based on the Body Dissatisfaction Score (PFRS-BD), most participants perceived themselves as heavier than their actual body size, a pattern observed in both heathy pregnant and lactating women (*p* > 0.05). However, when assessed using the Body Image Disturbance score (PFRS-BID), participants perceived themselves as slimmer than would be expected according to their actual BMI (*p* > 0.05). Although both groups showed similar trends in PFRS-BD and PFRS-BID scores, significant differences in overall body image scores were found between healthy pregnant and lactating women (*p* < 0.05).

The relationships among the NKLS, FFPLQ, EEQ, and PFRS are shown in Table 3. For pregnant women, significant differences were found in the NKLSA-food preference knowledge levels across their PFRS-related body dissatisfaction groups. The analysis revealed that a majority of pregnant women with a moderate level of food preference perceived themselves as being fatter than their actual weight was (*p* < 0.05). In the case of lactating women, NKLSA-based nutrition knowledge levels significantly differed among their FFPLQ levels and emotional eating groups. Notably, lactating women with moderate basic nutrition levels were at least low emotional eaters and had moderate knowledge regarding false facts about nutrition during pregnancy and lactation (*p* < 0.05). Additionally, PFRS-related body dissatisfaction significantly varied according to FFPLQ level, with lactating women who had moderate FFPLQ scores perceiving themselves as fatter than they were (*p* < 0.05).

**Table 3:** Relationships between nutritional knowledge level, false facts about nutrition during pregnancy and lactation, emotional eating and body image

	Pregnant (*n* = 100)						Lactate (*n* = 100)			
	NKLSA-Basic Nutrition									
		Low	Moderate	Good		Very Good	Low	Moderate	Good	Very Good
		**n (%)**	**n (%)**	**n (%)**		**n (%)**	**n (%)**	**n (%)**	**n (%)**	**n (%)**
EEQ	**Not an emotional eater**	12 (30.8)	25 (64.1)	1 (2.6)		1 (2.6)	12 (34.3)	17 (48.6)	6 (17.1)	0 (0)
	**Low emotional eater**	4 (13.4)	21 (72.4)	4 (13.8)		0 (0)	11 (30.6)	23 (63.9)	2 (5.6)	0 (0)
	**Emotional eater**	2 (6.7)	25 (83.3)	3 (10.0)		0 (0)	5 (17.9)	19 (67.9)	2 (7.1)	2 (7.1)
	**High emotional eater**	1 (50.0)	1 (50.0)	0 (0)		0 (0)	0 (0)	0 (0)	1 (100)	0 (0)
p-value	0.202						0.029*			
	**NKLSA-Basic Nutrition**									
		**Low**	**Moderate**	**Good**		**Very Good**	**Low**	**Moderate**	**Good**	**Very Good**
		**n (%)**	**n (%)**	**n (%)**		**n (%)**	**n (%)**	**n (%)**	**n (%)**	**n (%)**
FFPLQ	**Moderate**	15 (22.7)	45 (68.2)	5 (7.6)		1 (1.5)	23 (31.9)	43 (59.7)	6 (8.3)	0 (0)
	**Good**	4 (11.8)	27 (79.4)	3 (8.8)		0 (0)	5 (17.9)	16 (57.1)	5 (17.9)	2 (2)
p-value	0.499				0.041*					
	**NKLSA-Food Preference**									
		**Low**	**Moderate**	**Good**		**Very Good**	**Low**	**Moderate**	**Good**	**Very Good**
		**n (%)**	**n (%)**	**n (%)**		**n (%)**	**n (%)**	**n (%)**	**n (%)**	**n (%)**
PFRS Body Dissatisfaction	**No body dissatisfaction**	4 (44.4)	3 (33.3)	1 (11.1)		1 (11.1)	3 (21.4)	8 (57.1)	2 (14.3)	1 (7.1)
	**Slimmer than what they were**	3 (37.5)	2 (25.0)	3 (37.5)		3 (37.3)	2 (40.0)	1 (20.0)	2 (40)	0 (0)
	**Fatter than what they were**	8 (9.6)	44 (53.0)	20 (24.1)		20 (24.0)	5 (6.2)	41 (50.6)	21 (25.9)	14 (17.3)
p-value		0.036*					0.080			
	**FFPLQ**									
		**Moderate**		**Good**			**Moderate**		**Good**	
		**n (%)**		**n (%)**			**n (%)**		**n (%)**	
PFRS Body Dissatisfaction	**No body dissatisfaction**	5 (55.6)		4 (44.4)			13 (92.9)		1 (7.1)	
	**Slimmer than what they were**	8 (100)		0 (0)			5 (100)		0 (0)	
	**Fatter than what they were**	53 (63.9)		30 (36.1)			54 (66.7)		27 (33.3)	
p-value		0.094					0.047*			

_*Chi−square test, *p*<0.05_

Linear regression analysis of the NKLS, EEQ, FFPLQ, and PFRS is presented in Table 4. According to the analysis, EEQ was positively correlated with NKLS-basic nutrition (r: 0.288, *p* < 0.05) and NKLS-food preference. Moreover, food preferences were found to be a predictor of EEQ among pregnant and lactating women (Adj. R: 0.035, Adj. R: 0.030; respectively). For lactating women, the EEQ was positively correlated with the NKLS-food preference (*r* = 0.355, *p* < 0.05). Additionally, the FFPLQ was a predictor of body dissatisfaction (Adj. R: 0.062).

**Table 4:** Linear regression analysis of nutritional knowledge, emotional eating, misconceptions about nutrition and body dissatisfaction

	NKLSA-Basic Nutrition				NKLSA-Food Preference				FFPLQ			
	*r*	β	95% CI	*p*-value	*r*	β	95% CI	*p*-value	*r*	β	95% CI	*p*-value
Pregnant women-EEQ	**0.288**^**σ**^	0.102	−0.120-0.374	0.310	**0.355**^**σ**^	0.215	0.021–0.458	**0.032**^**φa**^	**-**	**-**	**-**	**-**
Lactating women- EEQ	-	-	-	-	**0.200**^**σ**^	0.200	0.004–0.475	**0.046**^**φb**^	**-**	**-**	**-**	**-**
Lactating women-PFRS- Body Dissatisfaction	-	-	-	-	-	-	-	-	0.134	0.268	0.015–0.094	**0.007**^**φc**^

_φ linear regression, *p*<0.05_

_σ Spearman’s rho, *p*<0.05_

_a: R: 0.215, R_^2^: _0.046, Adj. R: 0,035; b: R: 0.200, R_^2^: _0.040, Adj. R: 0,030; c: R: 0.278, R_^2^: _0.072, Adj. R: 0,062_

## Discussion

Pregnancy and lactation are unique and crucial periods for both maternal and fetal health. The study revealed that most pregnant and lactating women had moderate nutritional knowledge; however, they exhibited low levels of emotional eating behavior overall. Additionally, both groups experienced body dissatisfaction, often perceiving themselves as being fatter than they were.

In this study, 72% of pregnant women and 59% of lactating women presented moderate basic nutritional knowledge. Similarly, 49% of pregnant women and 50% of lactating women reported having knowledge about food preferences. The results from the FFPLQ indicated that the majority of the participants were aware of false facts and that 66% of pregnant and 72% of lactating women had moderate knowledge about nutrition. Similarly, Gezimu et al. [16] investigated knowledge, attitudes, practices, and determinants of nutrition during pregnancy and reported that 39.1% of participants were not knowledgeable about nutrition. Another study reported that only 33.3% of pregnant women passed a nutritional knowledge questionnaire [2]. Alemu Zerfu and Biadgiligin [68] reported that 47% of pregnant women lacked awareness of nutritional balance and diversity. Additionally, a study indicated that only 5% of pregnant women achieved 80% of the nutritional knowledge questionnaire score [69]. Another examination of maternal nutritional knowledge during lactation revealed that 52% of women had good nutritional knowledge [70]. Krishnendu and Devaki [71] reported that 70.8% of lactating women had moderate nutritional knowledge. Our results suggest that Turkish heathy pregnant and lactating women have higher levels of nutritional knowledge compared to those reported in other studies [2, 16, 68, 69]. These relatively higher levels of nutritional knowledge observed among Turkish pregnant and lactating women may be attributed to their higher educational background, increased access to health information, and the structured maternal health monitoring system in Türkiye, which facilitates more frequent exposure to nutrition-related counseling. These findings highlight the need for structured interventions to further enhance maternal nutritional literacy. To address the gaps identified in this study, targeted nutrition education programs should be integrated into routine antenatal and postnatal care services. Additionally, the development of culturally appropriate educational materials and strengthening of counseling practices delivered by healthcare professionals could help reinforce accurate nutritional information and dispel common misconceptions. Future community-based initiatives and multidisciplinary collaborations may also support women in translating nutritional knowledge into practice, thereby promoting healthier pregnancy and lactation outcomes.

In this study, 39% of pregnant and 35% of lactating women were nonemotional eaters. These findings suggest that a majority of participants engaged in emotional eating during pregnancy and lactation. Fariandini et al. [27] reported that 42.9% of pregnant women exhibited emotional eating patterns. Another study indicated that 25.1% of second-trimester pregnant women and 22.1% of third-trimester pregnant women practiced emotional eating [72]. Research on emotional eating during pregnancy and lactation is limited, but our findings are consistent with the literature. Hormonal changes and stress during these periods may affect eating patterns, as food cravings and increased energy expenditure can lead to snacking behaviors. Collectively, these factors may trigger emotional eating during pregnancy and lactation. The importance of adequate, healthy, and balanced nutrition should be emphasized among pregnant and lactating women, and stress management techniques may be beneficial for mitigating stress.

The study also revealed that pregnant and lactating women experienced body dissatisfaction and impaired body image. Most participants believed that they were slimmer than they actually were concerning body image, yet they thought they were fatter than they were concerning body dissatisfaction. Similarly, Chan et al. [36] reported that body dissatisfaction increased from prepregnancy to six weeks postpartum. A systematic review and meta-analysis indicated that women often have unrealistic expectations for their postpartum bodies [27]. Furthermore, another study showed that body image is closely related to postpartum depression in pregnant women [38] and that body dissatisfaction can result from shorter breastfeeding durations [37]. Body dissatisfaction tends to increase after childbirth due to changes in body appearance [41, 73, 74]. A study revealed that mothers frequently struggle to accept their postpartum bodies and fear that they would not return to their previous appearance [39]. Our findings are similar to the literature, emphasizing the need for adequate, healthy, and balanced nutrition from the beginning of pregnancy through the end of breastfeeding to ensure healthy weight gain and adequate nutrient intake during these periods.

Moreover, the study revealed a positive correlation between emotional eating and nutritional knowledge during both pregnancy and lactation. Considering the documented positive effects of nutrition education interventions on pregnant women’s dietary behaviors, it has been suggested that inadequate eating habits during pregnancy may be associated with insufficient nutritional knowledge [75]. In contrast, another study concluded that nutritional knowledge was not a predictor of emotional eating and indicated no relationship between the two [76]. However, research examining the nutritional literacy of pregnant women and aiming to enhance it through a targeted nutrition education intervention has shown that a comprehensive nutritional literacy program delivered in early pregnancy significantly improved women’s nutritional literacy in both the short and long term, optimized dietary behaviours, enhanced overall diet quality, and effectively supported appropriate gestational weight gain [77]. These conflicting results highlight the complexity of the relationship between emotional eating and nutritional knowledge. Our findings indicate that as food preference knowledge levels increase, so does emotional eating during pregnancy and lactation. The desire for optimal prenatal and postnatal outcomes may compel pregnant and lactating women to adopt healthier eating habits while seeking accurate nutritional information. This pursuit may inadvertently heighten anxiety and stress regarding healthy eating, potentially leading to increased emotional eating behaviors.

The study also demonstrated that body image was positively associated with nutritional knowledge during pregnancy, whereas knowledge of false nutritional facts served as a predictor of body dissatisfaction during lactation. Research has indicated that poorer body image is associated with a higher prevalence of restrained eating among healthy weight pregnant women [78]. Additionally, Easter et al. [79] reported that 25% of pregnant women exhibited poor eating behaviors due to concerns about body image and weight gain. Studies have further suggested that pregnant women with eating disorders may be particularly focused on weight gain and body image problems, increasing their vulnerability to psychiatric disorders [80]. A review noted that body image, dietary restrictions, and depression are strongly correlated with body dissatisfaction during pregnancy [81]. Our findings support the opinion that nutritional knowledge can be influenced by body image perceptions during pregnancy and that false nutritional information can impact body image during lactation. Thus, awareness of proper nutrition and reliable health information sources is vital to support women throughout pregnancy and lactation.

While this study has strengths, it also has limitations. One significant limitation is its cross-sectional design, which includes data from only a single center. Although the FFPLQ was approved based on its relevance, it was developed by researchers and lacks a formal validity study. Therefore, the results derived from the FFNPQ should be interpreted with caution, as the lack of validation may influence the reliability and generalizability of the findings. Nevertheless, this study is noteworthy, as it is one of the first to compare emotional eating, nutritional knowledge, misconceptions about nutrition and body image among pregnant and lactating women. Valid and reliable questionnaires were employed in the research, which also included participants from both groups.

## Conclusion

In conclusion, pregnancy and lactation represent critical periods for both mothers and their infants. Adequate, balanced, and healthy nutrition is essential for the well-being of both, with nutritional knowledge serving as a key factor in achieving this goal. This study highlights that nutritional knowledge can influence mothers’ emotional eating patterns and body image concerns during these stages. To increase the quality of life and nutritional status of pregnant and lactating women, efforts should be made to improve their nutritional knowledge.

### Recommendations

Based on the study’s findings, public health strategies, such as structured nutrition education programs emphasizing adequate, balanced, and healthy nutrition during pregnancy and lactation, should be implemented to improve nutritional knowledge among pregnant and lactating women.

## Supplementary Information

Supplementary Material 1.

## Data Availability

The data that support the findings of this study are available from the corresponding author, Hatice Kübra Barcın Güzeldere, upon reasonable request.

## Abbreviations

BMI: Body Mass Index
EEQ: Emotional Eating Questionnaire
FFPLQ: False Facts During Pregnancy and Lactation Questionnaire
NKLS: Nutrition Knowledge Level Determination Scale for Adults
PFRS: Photographic Figure Rating Scale
PFRS-BD: Body Dissatisfaction Score
PFRS-BID: Body Image Disturbance Score
