# Supplementary material for: Relationship between nutrition knowledge level, body perception and emotional eating among pregnant and lactating women: a cross-sectional study of 200 Turkish women
Source: BMC Pregnancy Childbirth. 2026 Jan 24;26:173. doi: 10.1186/s12884-025-08622-9 (PMC12910812; doi:10.1186/s12884-025-08622-9)
Supplement: Supplementary file 1 — Supplementary Material 1. [file 12884_2025_8622_MOESM1_ESM.docx]

**THE EFFECT OF NUTRITIONAL AWARENESS LEVELS OF WOMEN IN PREGNANCY AND LACTATION ON BODY IMAGE AND EMOTIONAL EATING**

SURVEY NO: …………

NAME-SURNAME: ……………………………

**PART I. DEMOGRAPHIC DATA**

1. Age: ………….

2. Weight: …………

3. Height: ………….

4. Your profession: …………………..

5. Please mark your educational status.

A. I do not know how to read and write B. Primary school C. Secondary school D. High school E. University F. Master's degree G. PhD

6. How do you evaluate your economic situation?

A. Low B. Medium C. Good

7. What was the age you learned about your pregnancy?

A. 18-20 B. 20-30 C. 30-40 D. 40-50

8. How much weight did you gain during your pregnancy?

A. 0-5 B. 5-10 C. 10-15 D. 15-20 E. 20 and above

9. Pregnancy week/Lactation period:…………

10. Do you have any disease?

A. Yes B. No

11. If your answer is yes, please write your disease……….

12. If you use a medication regularly, please write the name of your medication……..

13. Did you have cravings (wanting to eat a lot of food) during your pregnancy?

A. Yes B. No

14. If you had cravings, which foods did you crave the most? (You can choose more than one option).

A. Sugar added foods (chocolate, cake, cookies, cakes, etc.)

B. Sugar added drinks (ready-made fruit juices, hot chocolate, ready-made lemonade, iced tea, coffees with sugar and cream)

C. Carbonated drinks (cola, soda, fruit soda)

D. Homemade fresh fruit juices (including lemonade)

E. Fast-food foods (hamburger, pizza, pita, lahmacun, döner, etc.)

F. Sour fruits (tangerine, orange, kiwi, plum, etc.)

G. Sweet fruits (watermelon, melon, peach, strawberry, etc.)

H. Sour foods (pickles, etc.)

I. Salty foods (cheese, salty crackers, etc.)

J. Ayran

15. When you evaluate your diet, do you think you eat healthy?

A. Yes B. No

16. How many meals do you eat per day?

A. 1 B. 2 C. 3 D. 4 E. 5 F. 6 G. 7 and above

17. Please mark which meals you consume.

A. Breakfast B. Mid-morning C. Lunch D. Afternoon E. Evening F. Night

18. If you skip a meal during the day, which meal(s) do you skip?

A. Breakfast B. Mid-morning C. Lunch D. Afternoon E. Evening F. Night

**PART II. NUTRITIONAL KNOWLEDGE LEVEL FOR ADULTS SCALE (NKLAS)**

**BASIC NUTRITION AND NUTRITION-HEALTH INFORMATION**

|  | **Strongly agree** | **Agree** | **Neighter agree or disagree** | **Disagree** | **Strongly disagree** |
| --- | --- | --- | --- | --- | --- |
| 1. Natural, freshly squeezed fruit juices do not contain sugar. |  |  |  |  |  |
| 1. Carrots are a good source of vitamin A. |  |  |  |  |  |
| 1. Vitamins and minerals provide energy. |  |  |  |  |  |
| 1. **Carbohydrates are the main source of energy** |  |  |  |  |  |
| 1. The nutritional value of frozen products is lower than fresh foods. |  |  |  |  |  |
| 1. Fruits have high protein content. |  |  |  |  |  |
| 1. Eggs and red meat are similar in terms of the amount of protein they contain. |  |  |  |  |  |
| 1. **Olive oil consumption increases cholesterol.** |  |  |  |  |  |
| 1. Dry bean salad has a high fiber content. |  |  |  |  |  |
| 1. Fats found in processed meat products such as salami and sausages are harmful to health. |  |  |  |  |  |
| 1. Calcium mineral found in milk and dairy products is important for bone and dental health. |  |  |  |  |  |
| 1. The best source of vitamin D, which is necessary for protection against osteoporosis, is the sun. |  |  |  |  |  |
| 1. Vitamin E is a very effective vitamin for vision. |  |  |  |  |  |
| 1. Vitamin C found in oranges strengthens immunity and protects against colds and flu infections. |  |  |  |  |  |
| 1. Consuming whole grain (brown) bread is beneficial for the nervous system due to the vitamins it contains |  |  |  |  |  |
| 1. Excessive consumption of salt does not affect blood pressure. |  |  |  |  |  |
| 1. Red meat is effective in preventing forgetfulness because it contains vitamin B12. |  |  |  |  |  |
| 1. Red and purple vegetables and fruits are protective against cancer. |  |  |  |  |  |
| 1. Fish has a higher saturated fat content than red meat. |  |  |  |  |  |
| 1. Fats contain less energy than proteins and carbohydrates. |  |  |  |  |  |

******* **What is the degree of relationship between nutrition and health? Please evaluate.**

| **1** | **2** | **3** | **4** | **5** | **6** | **7** | **8** | **9** | **10** |
| --- | --- | --- | --- | --- | --- | --- | --- | --- | --- |
| ← no relationship high relationship → | | | | | | | | | |

**FOOD PREFERENCE**

|  | **Strongly agree** | **Agree** | **Neighter agree or disagree** | **Disagree** | **Strongly disagree** |
| --- | --- | --- | --- | --- | --- |
| 1. It is healthier for diabetics to consume the fruit itself (without peeling it if possible) instead of fruit juice. |  |  |  |  |  |
| 1. Consuming fiber containing foods instead of sugar added food prevents from constipation. |  |  |  |  |  |
| 1. An individual who wants to reduce the amount of fat they consume with food should prefer grilled chicken instead of fried chicken. |  |  |  |  |  |
| 1. A person who wants to increase the protein intake in a meal should prefer egg spinach dish instead of bulgur spinach dish. |  |  |  |  |  |
| 1. It is a better choice to consume whole wheat crackers instead of sweet biscuits as a snack. |  |  |  |  |  |
| 1. **Its more benefical to put 3-4 dried apricots in children’s lunch boxes instead of wafers.** |  |  |  |  |  |
| 1. It is more correct for an adult to meet their fluid needs by drinking water instead of drinks such as tea and coffee. |  |  |  |  |  |
| 1. It is more beneficial to get vitamins and minerals from medicines instead of taking them directly from food. |  |  |  |  |  |
| 1. Proteins in animal-based foods (such as meat, fish, milk, eggs) are very important for body health. |  |  |  |  |  |
| 1. White bread is healthier than whole grain (brown) bread. |  |  |  |  |  |
| 1. In order to reduce salt intake, cabbage salad should be preferred instead of sauerkraut |  |  |  |  |  |
| 1. Someone who wants to reduce the amount of fat they take from food can prefer light milk. |  |  |  |  |  |

***How ​​accurate do you find the food choices you make in your daily life? Please evaluate.

| **1** | **2** | **3** | **4** | **5** | **6** | **7** | **8** | **9** | **10** |
| --- | --- | --- | --- | --- | --- | --- | --- | --- | --- |
| ← inadequate adequate → | | | | | | | | | |

**PART III. QUESTIONS ABOUT NUTRITION DURING PREGNANCY/LACTATION**

1. Vitamin A supplements should definitely be used during pregnancy.

A. Yes B. No

2. Folic acid supplements should definitely be used during pregnancy.

A. Yes B. No

3. Pregnant women should not eat fish.

A. Yes B. No

4. Pregnant women should eat for two.

A. Yes B. No

5. Nettle tea increases milk during lactation.

A. Yes B. No

6. Caffeine consumption causes premature birth.

A. Yes B. No

7. Echinacea tea should be preferred during pregnancy.

A. Yes B. No

8. Drinking herbal tea during pregnancy is harmful.

A. Yes B. No

9. Unlimited green tea can be drunk during pregnancy.

A. Yes B. No

10. B6 supplements can be taken to suppress nausea during pregnancy.

A. Yes B. No

11. During lactation, mothers can drink malt beverages to increase milk.

A. Yes B. No

12. Meat consumption is important for vitamin D intake during pregnancy and lactation.

A. Yes B. No

13. Iodized salt should not be used.

A. Yes B. No

14. If a lactating mother has gas problems, the mother should definitely not drink milk.

A. Yes B. No

15. If a lactating mother has gas problems, the mother can drink lactose-free milk.

A. Yes B. No

16. Pregnant women should consume eggs every day.

A. Yes B. No

17. During lactation, they should consume at least 2.5 L of water per day.

A. Yes B. No

18. During lactation, foods such as eggs, milk, and yogurt should be consumed to meet daily protein and calcium needs.

A. Yes B. No

19. During pregnancy, nuts and walnuts can be consumed for the development of the baby's nervous system.

A. Yes B. No

20. During pregnancy, I should only consume high-energy foods for my baby to gain weight.

A. Yes B. No

21. Drinking boza during pregnancy helps my baby gain weight.

A. Yes B. No

22. Plenty of soda should be drunk during pregnancy.

A. Yes B. No

23. Drinking milk during pregnancy and lactation causes anemia.

A. Yes B. No

24. Processed products such as salami, soudjouk, sausage and pastrami should not be consumed.

A. Yes B. No

25. Alcohol can be consumed during pregnancy.

A. Yes B. No

**Part IV. EMOTİONAL EATİNG QUESTİONNAİRE (EEQ)**


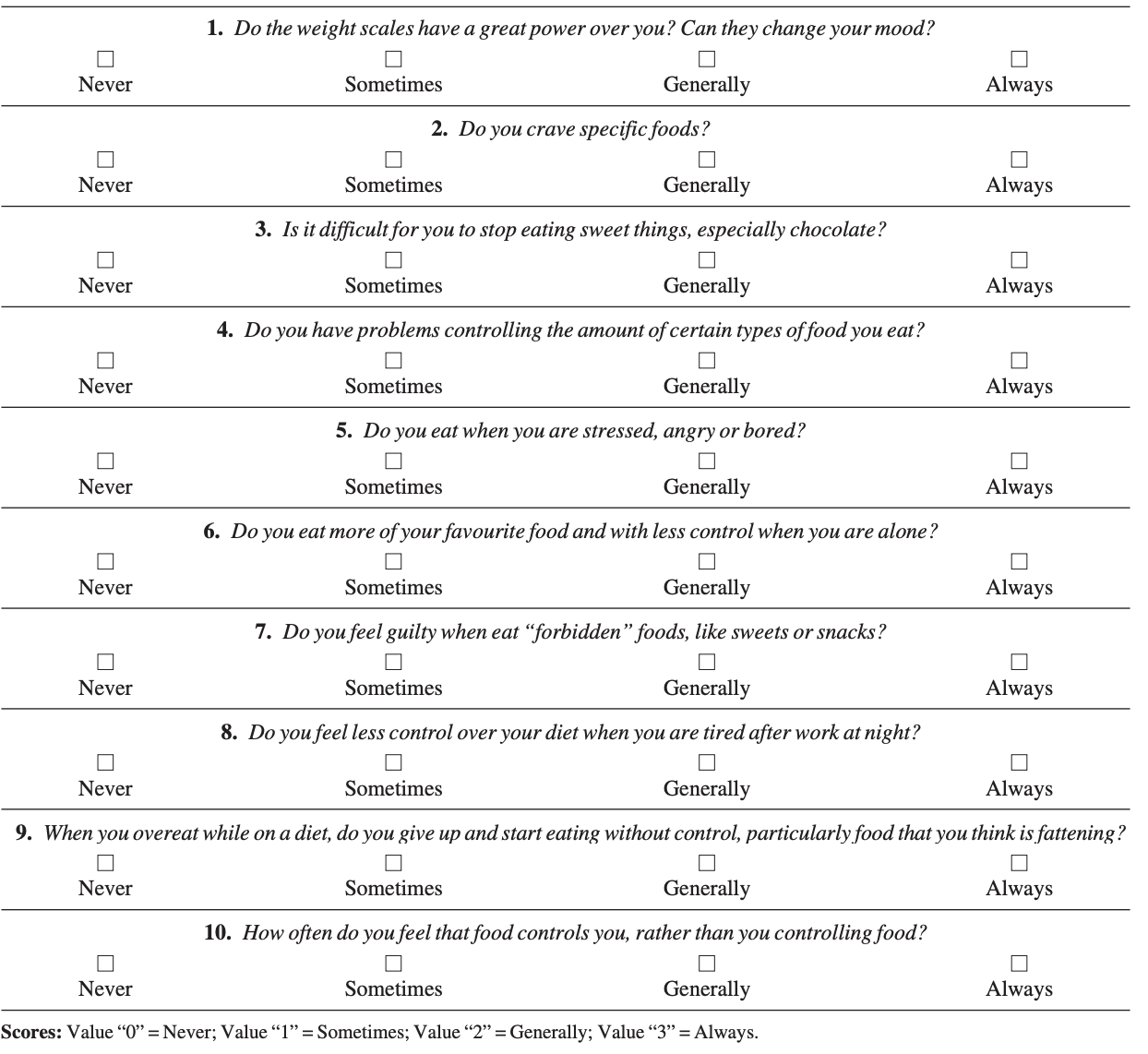


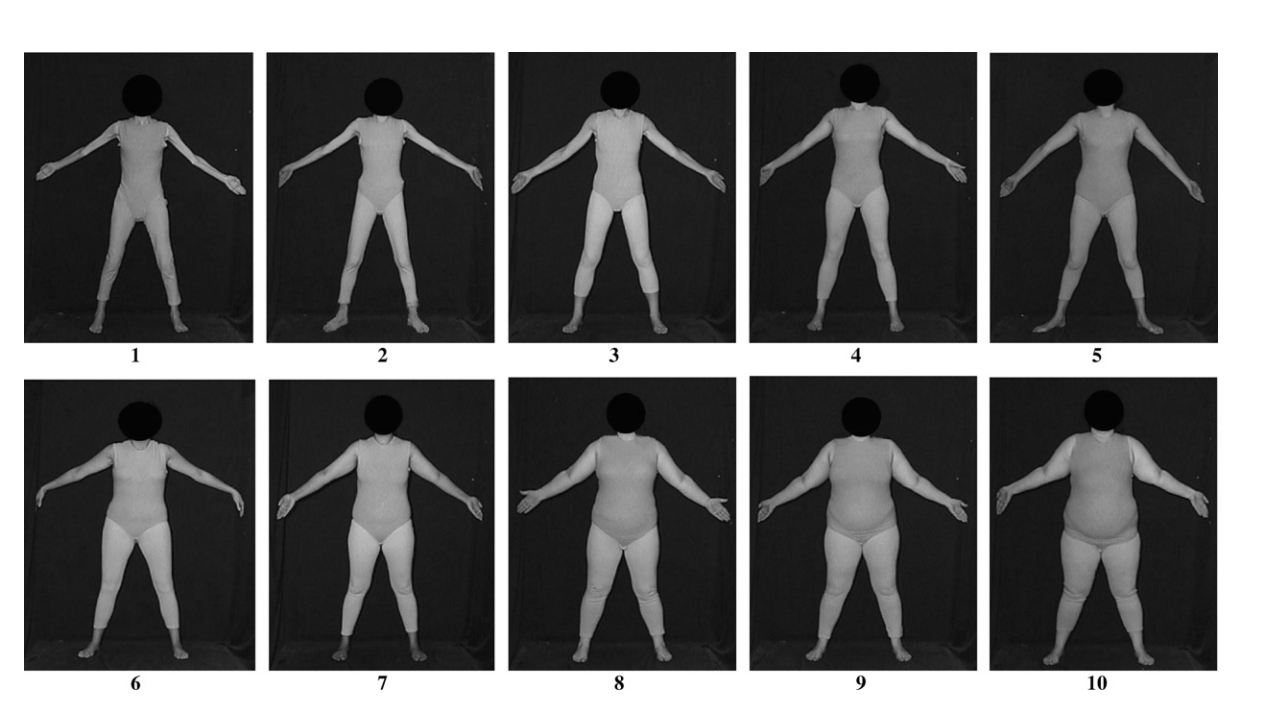


**FORM 1: Please answer the following questions:**

1. Which picture do you find most physically attractive? ............

2. Which picture do you find most physically attractive? ............

3. Which picture do you find most physically attractive? .............

4. Which picture most resembles your current body type? ............

5. Which picture most resembles the body type you would most like to have? .........

**Please use the rating scale below to answer the following question:**

1---------2------------3---------4---------5---------6---------7---------8----------9

Not at all Very little Somewhat Quite a bit

**How physically attractive do you find each of the women you see in the pictures above?**

Picture 1 …… Picture 2 …… Picture 3 …… Picture 4 …… Picture 5 ……

Picture 6 …… Picture 7 …… Picture 8 …… Picture 9 …… Picture 10 ……
